# Effect of SARS-CoV-2 mRNA vaccination on ocular herpes simplex and varicella-zoster virus reactivation: should preventive antiviral treatment be given in known herpes patients

**DOI:** 10.1186/s12348-021-00262-2

**Published:** 2021-09-17

**Authors:** Carl P. Herbort, Ioannis Papasavvas

**Affiliations:** Inflammatory and Retinal Eye Diseases, Centre for Ophthalmic Specialised Care (COS), Rue Charles-Monnard 6, 1003 Lausanne, Switzerland

To the editor.

Herpesviruses, including herpes simplex virus 1 (HSV-1) and varicella-zoster virus (VZV) are commensal viruses of humans since millenaries.

Close to 80% of adults in temperate countries are exposed to HSV-1 and this proportion reaches more than 97% in case of VZV [1].

After primoinfection these viruses remain in a latent form in sensory neuronal ganglia. The colonisation of the ganglia, including the trigeminal ganglion, occurs by progression of the virus mainly within peptidergic neurons [2]. Latency is an active and complex immune mechanism where CD8-positive T cells play an important role [3]. Although mRNA vaccines stimulate CD8+ T cells it could be hypothesised that it might dysregulate latency mechanisms in the sensory nerve ganglions [4]. In case of sarcoidosis when T cells are monopolised in the granulomas there is rescue humoral compensation to control herpes viruses by polyclonal anti-herpes antibody activation [5]. In case of transient or prolonged dysfunction of the immune system by diverse causes, reactivation of the virus can occur. It then reinfects peripheral sites by travelling down neurons causing herpes labialis, dendritic keratitis, herpetic uveitis or shingles in case of VZV virus. A recent study reported 14/414 (3.4%) cases of herpes simplex and varicella-zoster eruptions among cutaneous findings after mRNA-based vaccinations, a relatively large percentage [6]. However, no details were given on these patients. We recently reported on three cases of herpes zoster ophthalmicus (HZO) after mRNA anti-SARS-CoV-2 vaccinations (Moderna and Pfizer BioNTech respectively), 2 weeks after first dose vaccination in two patients and 2 weeks after a Pfizer Bio NTech booster vaccination in another patient who had undergone Covid-19 infection [7]. Non ocular varicella-zoster reactivations after mRNA vaccinations have recently been reported [8].

## Case report

**Fig. 1:**
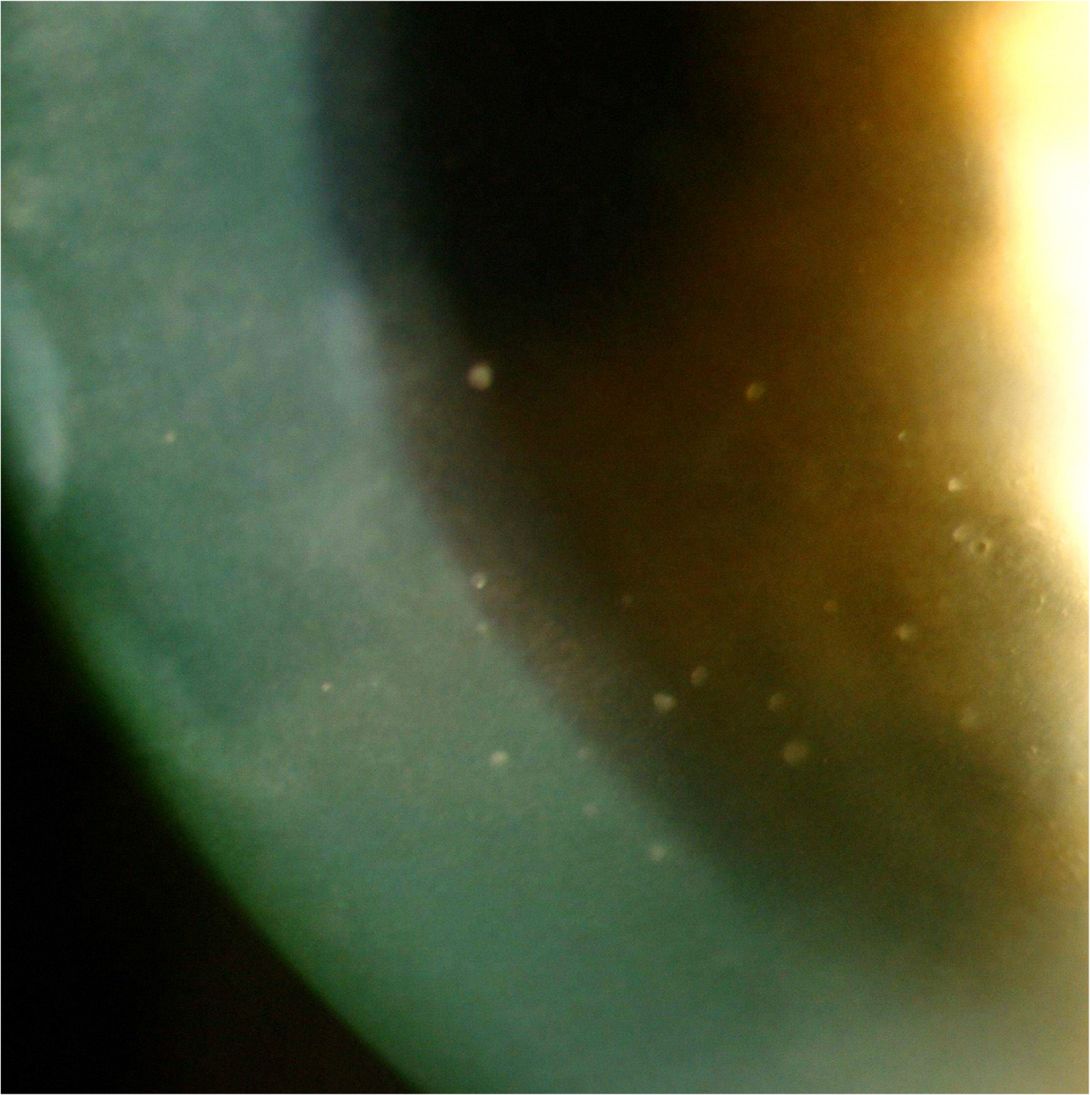
Herpetic kerato-uveitis: Numerous granulomatous KPs on the endothelium

We recently examined a 53-year-old man, treated in the past for herpes kerato-uveitis OD that had been inactive for 18 months without treatment. Five days after the second dose of Moderna vaccine, he presented with a severe flare-up of a granulomatous hypertensive uveitis in his right eye with numerous granulomatous KPs and an increased pressure of 41 mmHg (Fig. 1). Under valacyclovir (500 mg QID), 0.1% dexamethasone drops QID and dorzolamide hydrochloride – timolol maleate eye drops BID, pressure normalised within 6 days, laser flare photometry (LFP) flare, decreased from 19.4 ph/ms to 13.2 ph/ms 3 weeks later and granulomatous KPs had almost disappeared. Because of fluctuating IOP and LFP values thereafter, Valacyclovir (500 mg BID), Acetazolamide (125 mg/day), dorzolamide hydrochloride BID and dexamethasone 0.1% eye drops once daily had to be maintained until the last recorded follow-up two and a half months later with LFP values of 12.2 ph/ms and an IOP value of 16 mmHg.

It is difficult to know whether this episode was purely inflammatory. Even if this was the case dual corticosteroid and antiviral therapy has to be given in such circumstances.

Since this case, we advise patients having had several herpes uveitis episodes to remain under valacyclovir therapy during vaccination despite quiet uveitis to avoid potential reactivation, which was undertaken for two patients so far.

## Comment

HSV-1 and VZV infections can cause substantial ocular morbidity. Since the availability of efficient antiviral agents, management of eye disease caused by herpes viruses are successfully treated in most cases and complications have been reduced [9]. However, each new episode can harm eye structures. Moreover, some herpes reactivations such as acute retinal necrosis are much more deleterious and their management much more difficult. Therefore, in patients having presented herpesvirus infections in the past, the question can be raised whether preventive antiviral therapy should be recommended, possibly at least in high-risk patients, in case of mRNA vaccination after discussing it with the patient.

## Data Availability

For data, please refer to corresponding author.

## Abbreviations

HSV-1: Herpes simplex virus 1
VZV: Varicella-zoster virus
CD8+ T cells: Cluster of differentiation 8 positive T cells
mRNA: Messenger ribonucleic acid
HZO: Herpes zoster ophthalmicus
